# Exercise Training Mitigates Water Pipe Smoke Exposure-Induced Pulmonary Impairment via Inhibiting NF-*κ*B and Activating Nrf2 Signalling Pathways

**DOI:** 10.1155/2018/7459612

**Published:** 2018-03-06

**Authors:** Abderrahim Nemmar, Suhail Al-Salam, Priya Yuvaraju, Sumaya Beegam, Badreldin H. Ali

**Affiliations:** ^1^Department of Physiology, College of Medicine and Health Sciences, United Arab Emirates University, P.O. Box 17666, Al Ain, UAE; ^2^Department of Pathology, College of Medicine and Health Sciences, United Arab Emirates University, P.O. Box 17666, Al Ain, UAE; ^3^Department of Pharmacology and Clinical Pharmacy, College of Medicine & Health Sciences, Sultan Qaboos University, P.O. Box 35, Muscat 123, Al Khoudh, Oman

## Abstract

Water pipe smoking is a tobacco smoking method commonly used in Eastern countries and is gaining popularity in Europe and North America, in particular among adolescents and young adults. Several clinical and experimental studies have reported that exposure to water pipe smoke (WPS) induces lung inflammation and impairment of pulmonary function. However, the mechanisms of such effects are not understood, as are data on the possible palliative effect of exercise training. The present study evaluated the effects of regular aerobic exercise training (treadmill: 5 days/week, 40 min/day) on subchronic exposure to WPS (30 minutes/day, 5 days/week for 2 months). C57BL/6 mice were exposed to air or WPS with or without exercise training. Airway resistance measured using forced oscillation technique was significantly and dose-dependently increased in the WPS-exposed group when compared with the air-exposed one. Exercise training significantly prevented the effect of WPS on airway resistance. Histologically, the lungs of WPS-exposed mice had focal moderate interstitial inflammatory cell infiltration consisting of neutrophil polymorphs, plasma cells, and lymphocytes. There was a mild increase in intra-alveolar macrophages and a focal damage to alveolar septae in some foci. Exercise training significantly alleviated these effects and also decreased the WPS-induced increase of tumor necrosis factor *α* and interleukin 6 concentrations and attenuated the increase of 8-isoprostane in lung homogenates. Likewise, the lung DNA damage induced by WPS was significantly inhibited by exercise training. Moreover, exercise training inhibited nuclear factor kappa-B (NF-*κ*B) expression induced by WPS and increased that of nuclear factor erythroid 2-related factor 2 (Nrf2). Our findings suggest that exercise training significantly mitigated WPS-induced increase in airway resistance, inflammation, oxidative stress, and DNA damage via mechanisms that include inhibiting NF-*κ*B and activating Nrf2 signalling pathways.

## 1. Introduction

Water pipe smoking, known under various synonyms including hubble-bubble, shisha, hookah, or narghile, is a tobacco smoking method widely utilized in the Middle East, Turkey, India, and China and is at present gaining increasing popularity in Europe and North America, in particular among adolescents and young adults. [1–4]. The prevalence of current WPS in adults was found to reach 4 to 12% in Gulf countries, 15% in Lebanon, and 9 to 12% in Syria [5]. In North America, studies reported a prevalence of 8.8% in adults who have ever used WPS and 11.4% reported current WPS use [6, 7]. The popularity of WPS in adolescents and young adults is increasing at disturbing rates. For example, 43 to 61% of college students in the Middle East indicated lifetime WPS and 5 to 43% mentioned previous-month or present WPS use [8]. Moreover, it has been described that 1 out of 5 American and European college students reported previous-year WPS use while lifetime WPS was found to reach 15–41% and previous-month WPS rates went from 7 to 21% [9].

Studies on the adverse pulmonary effects of WPS are relatively scarce compared with those related to cigarette smoking. Clinical studies have reported that both acute and chronic WPS induce increase in COHb, blood pressure, and heart and respiratory rates and induced lung inflammation and impairment in pulmonary function [10–13]. Likewise, experimental studies have reported that subacute and chronic exposure to WPS induces lung inflammation, oxidative stress, and increase in airway resistance [14–16].

Although numerous antismoking campaigns are in place, many sufferers are left with permanent damage and need ongoing treatment, even after cessation of smoking. In fact, in view of the challenging tobacco-dependence syndrome, management requires various approaches, including compliance to medication and behavioral and other modalities of treatment [17]. Nevertheless, no study has been reported on how to reduce the pulmonary adverse effects of WPS. Exercise training is essential in averting and treating lung disorders. Moderate exercise training has been reported to improve cardiopulmonary capacity and airway immunity and protect the lungs against oxidative stress observed in lung diseases [18, 19]. As it is well recognized that exposure to WPS induces pulmonary inflammation and oxidative stress [14–16], we sought, here, to evaluate whether, and to what extent, can exercise training improve WPS-induced lung inflammation and oxidative stress and increase in airway resistance. Such study has, as far as we are aware, never been reported so far.

Here, the aim of this study was to assess the possible ameliorative effect of aerobic exercise training against WPS-induced pulmonary impairment and the mechanisms underlying these effects.

## 2. Material and Methods

### 2.1. Animals and Treatments

This project was evaluated and accepted by the Institutional Review Board of the United Arab Emirates University, College of Medicine and Health Sciences, and experiments were done in agreement with protocols agreed by the Institutional Animal Care and Research Advisory Committee.

### 2.2. WPS Exposure

C57BL/6 mice (Taconic Farms Inc., Germantown, NY, USA) were housed in a conventional animal house and kept on a 12-hour light-dark cycle (lights on at 6:00 a.m.). The animals were put in cages and provided with pelleted food and H_2_O ad libitum. Mice were allowed to adjust to the exposure structure for 1 week prior the beginning of exposure to WPS or air.

Animals were put in soft restraints and connected to the exposure tower [14, 15, 20–22]. The animals were exposed to either air or WPS via their noses by a nose-only exposure system linked to a water pipe (InExpose System, Scireq, Canada). Mice were exposed to a commercially available apple-flavored tobacco (Al Fakher Tobacco Trading, Ajman, United Arab Emirates). Tobacco was lit with an instant light charcoal disk (Star, 3.5 cm diameter and 1 cm width). As seen in human use, the smoke from the water pipe goes initially through the water before it was drawn into the exposure tower. The exposure regime is controlled by a computerized system (InExpose System, Scireq, Canada) [23]. A computer-controlled puff was produced every minute, leading to a 2 s puff period of WPS exposure followed by 58 s of air. The duration of an exposure session was 30 min/day [14, 15, 20, 21, 23]. The latter was chosen from a recent work that has evaluated the cardiorespiratory impact of WPS in human subjects [13]. Mice were exposed for 2 consecutive months (30 minutes/day, 5 days/week).

### 2.3. Exercise protocol

The exercise protocol used was similar to that reported before by Vieira et al. [24]. Animals were exercised during their active period, that is, 09:00 and 12:00 on an Exer 3/6 treadmill—Columbus motorized treadmill (Columbus Instruments, Columbus, OH, USA)—for 40 min/day at 12 m/min, 12% grade, 5 times/week for the duration of two months. The latter intensity coincides to 65–70% of maximal oxygen uptake [24, 25]. A foam sponge was put at the back of each treadmill lane to avoid the injury of the mice. All mice conformed to this exercise protocol. The interval and intensity were augmented gradually so that the mice were running at the set level by the 8 training session [24]. Control animals were exposed to the same noise and handling as the mice group which went through exercise training.

One hour following the end of the exercise training period, animals were exposed to WPS as described above. In total, 4 groups of mice were studied, that is, air (nonexercisers), WPS (nonexercisers), exercise + air, and exercise + WPS.

### 2.4. Airway Reactivity to Methacholine

Airway hyperreactivity responses were assessed using a forced oscillation technique (FlexiVent, SCIREQ, Montreal, Canada) as described before [15, 26, 27].

### 2.5. Histopathology

In separate animals, the heart-lung block was excised and fixed in 10% neutral formalin at a constant hydrostatic pressure of 20–25 cm fluid column for 24 hours [23, 28] which was followed by dehydration in increasing concentrations of ethanol, clearing with xylene and embedding with paraffin. Three *μ*m sections were prepared from paraffin blocks and stained with haematoxylin and eosin [29]. The stained sections were blindly evaluated by the histopathologist who participates in this project using light microscopy.

### 2.6. Measurement of Tumor Necrosis Factor (TNF*α*), Interleukin 6 (IL-6), and 8-Isoprostane

At the end of the two-month-exposure period to either WPS or air, with or without exercise training, individual mice were sacrificed by an overdose of sodium pentobarbital, and their lungs were quickly collected and rinsed with ice-cold PBS (pH 7.4) before homogenization, as described before [29, 30]. The homogenates were centrifuged for 10 min at 3000 ×g to remove cellular debris, and the supernatants were utilized for additional analysis [29]. Protein content was assessed by Bradford's technique. The concentrations of TNF*α* and IL-6 were measured using ELISA Kits (Duo Set, R & D systems, Minneapolis, MN, USA). 8-Isoprostane concentrations were evaluated using an ELISA Kit (Cayman Chemicals, Michigan, USA) [31].

### 2.7. DNA Damage Assessment by COMET Assay

In separate mice, the lungs of mice obtained from the various studied groups were used to quantify the DNA damage by COMET assay. The latter was evaluated as previously described [15, 32–35], and the assessment of length of the DNA migration (i.e., diameter of the nucleus plus migrated DNA) was measured using the image analysis AxioVision 3.1 software (Carl Zeiss, Canada) [15, 36].

### 2.8. Western Blot Analysis

Protein expressions for NF-*κ*B p65 and Nrf2 were assessed using Western blotting techniques [34]. Lung tissues collected from the mice were straightway snap frozen with liquid nitrogen and kept at −80°C. After that, the tissues were weighed, rinsed with saline, and homogenized with lysis buffer (pH 7.4) containing NaCl (140 mM), KCl (300 mM), Trizma base (10 mM), EDTA (1 mM), Triton X-100 0.5% (*v*/*v*), sodium deoxycholate 0.5% (*w*/*v*), protease, and phosphatase inhibitor [34]. The homogenates were centrifuged for 20 min at 4°C. The supernatants were collected, and protein quantification was performed with a Pierce bicinchoninic acid protein assay kit (Thermo Scientific) [34]. A protein sample (35 *μ*g) was electrophoretically isolated by 10% sodium dodecyl sulfate polyacrylamide gel electrophoresis and then transferred onto polyvinylidene difluoride membranes. The immunoblots were then blocked with 5% nonfat milk and then probed with either the rabbit monoclonal NF-*κ*B p65 antibody (1 : 25,000 dilution, Abcam) or rabbit monoclonal Nrf2 antibody (1 : 2000 dilution, Abcam) at 4°C overnight. The blots were then incubated with goat anti-rabbit IgG horseradish peroxidase conjugated secondary antibody (1 : 5000 dilution, Abcam) for 2 h at room temperature and developed using Pierce enhanced chemiluminescent plus Western blotting substrate Kit (Thermo Scientific). The densitometric analysis of the protein bands was performed for NF-*κ*B p65 and Nrf2 with Typhoon FLA 9500 (GE Healthcare Bio-Sciences AB, Uppsala, Sweden). Blots were then reprobed with mouse monoclonal GAPDH antibody (1 : 5000 dilution, Abcam) and used as a control.

### 2.9. Statistics

All statistical analyses were executed with GraphPad Prism Software version 5. Comparisons between the various groups were achieved by one-way analysis of variance (ANOVA), followed by Newman-Keuls multiple range tests. The data in figures were reported as mean ± SEM. *P* values < 0.05 are considered significant.

## 3. Results

### 3.1. Airway Hyperreactivity to Methacholine

**Figure 1:**
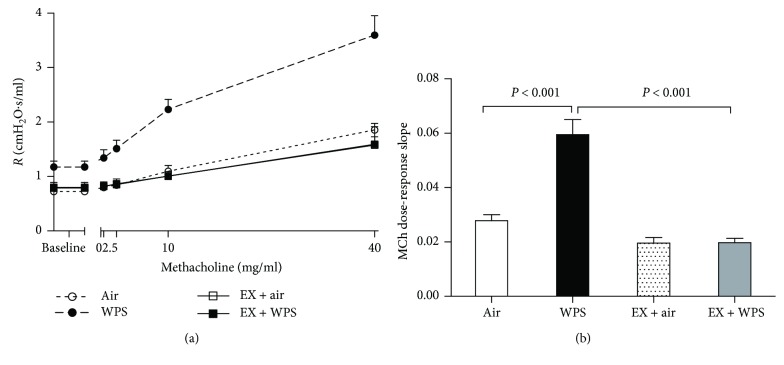
Airway hyperresponsiveness. The airway resistance (*R*), after increasing concentrations of methacholine (MCh) (0–40 mg/ml), was measured via the forced oscillation technique (FlexiVent), at the end of the 2-month-exposure period to water pipe smoke (WPS) or air with or without exercise training. Dose-response relationship of total respiratory system resistance to increasing doses of MCh (a). From the resistance MCh dose-response curve in (a), an index of airway responsiveness was calculated as the slope of the linear regression using 0–40 mg/ml concentrations (b). Data are mean ± SEM (*n* = 8–9).

Figure 1(a) illustrates the airway resistance, following increasing concentrations of methacholine (0–40 mg/ml), after exposure to WPS or air, with or without exercise training. The airway resistance was dose-dependently increased in the WPS group compared with the air-exposed group. No difference in airway resistance was seen between the air group and the exercise + air group. Remarkably, exercise training significantly inhibited the increase in airway resistance induced by WPS. From the resistance methacholine dose-response curve, an index of airway responsiveness was calculated as the slope of the linear regression using 0–40 mg/ml concentration (Figure 1(b)). The methacholine dose-response slope was significantly increased in the WPS group compared with the air group (*P* < 0.001), and this effect was significantly inhibited by exercise training (*P* < 0.001).

### 3.2. Lung Histopathology

**Figure 2:**
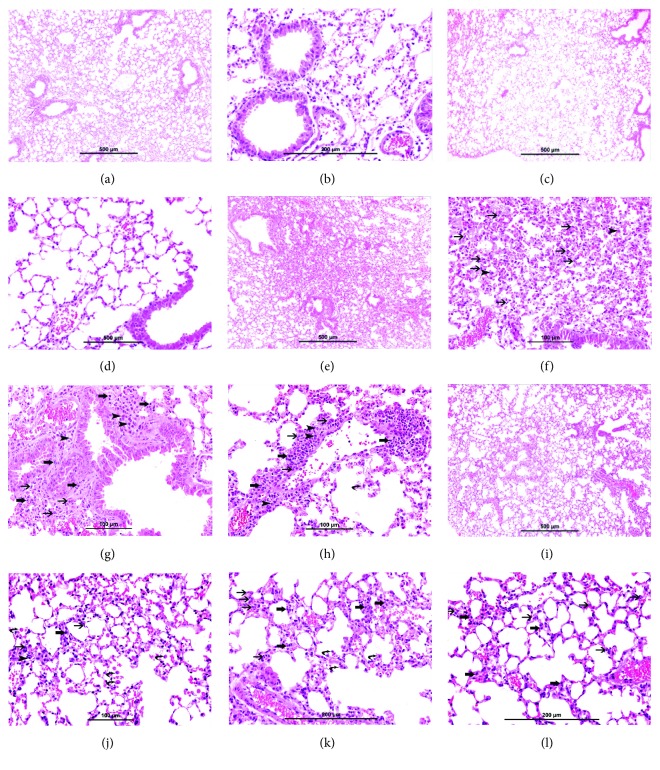
Representative light microscopy sections of lung tissues of mice, at the end of the 2-month-exposure period to water pipe smoke (WPS) or air with or without exercise training. (a, b) The air-exposed group shows normal lung tissue with unremarkable changes. (c, d) The exercise + air group shows normal lung tissue with unremarkable changes. (e–h) The WPS group: (e) low power view showing widening of interstitial space with mixed inflammatory cell infiltration. (f) shows moderate expansion of the alveolar interstitial space with many neutrophil polymorphs (thin arrow) and increased intra-alveolar macrophages (arrow head). (g) shows moderate expansion of the alveolar interstitial space with many neutrophil polymorphs (thin arrow), lymphocytes (thick arrow), and plasma cells (arrow head). (h) shows moderate expansion of the alveolar interstitial space with many neutrophil polymorphs (thin arrow), lymphocytes (thick arrow), and plasma cells (arrow head). There is a focal destruction of interalveolar septae (curved arrow). (i–l) The exercise + WPS group: (i) low power view showing focal mild widening of interstitial space with focal mild mixed inflammatory cell infiltration. (j–l) show focal mild expansion of the alveolar interstitial space with few neutrophil polymorphs (thin arrow) and few lymphocytes (thick arrow). There is a mild increase of alveolar macrophages (curved arrow).

The light microscopy analysis of the lung sections from air-exposed mice (Figures 2(a) and 2(b)) and the exercise + air mice (Figures 2(c) and 2(d)) showed normal lung tissue with unremarkable changes. The lung sections of WPS-exposed mice (Figures 2(e)–2(h)) showed focal moderate interstitial inflammatory cell infiltration consisting of neutrophil polymorphs, plasma cells, and lymphocytes. There was a moderate increase in intra-alveolar macrophages and focal damage to alveolar septae in some foci. In the exercise + WPS group (Figures 2(i)–2(l)), there was preservation of lung architecture with only few foci of mild interstitial inflammatory cell infiltration consisting of neutrophil polymorphs, lymphocytes, and very few plasma cells and mild increase in intra-alveolar macrophages.

### 3.3. TNF*α* and IL-6 in Lung Homogenate

**Figure 3:**
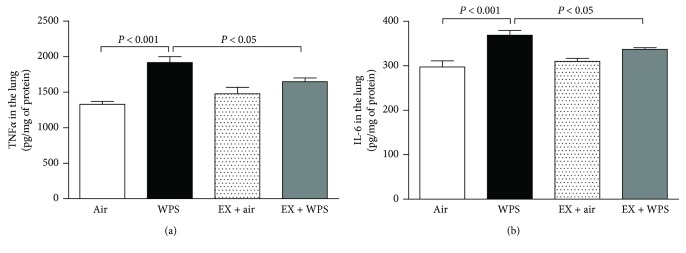
Tumor necrosis factor *α* (TNF*α*) (a) and interleukin 6 (IL-6) (b) concentrations in lung homogenate, at the end of the 2-month-exposure period to water pipe smoke (WPS) or air with or without exercise (EX) training. Data are mean ± SEM (*n* = 7–9 in each group).

The concentration of TNF*α* in lung homogenates was significantly increased following WPS exposure compared with the air-exposed group (*P* < 0.001; Figure 3(a)). Exercise training has significantly prevented the increase in TNF*α* concentration caused by WPS (Figure 3(a)). Likewise, IL-6 concentration was significantly increased by the subchronic exposure to WPS compared with the control group (Figure 3(b)). The latter augmentation of IL-6 was significantly abrogated in the exercise training + WPS group.

### 3.4. 8-Isoprostane Concentrations in Lung Homogenates

**Figure 4:**
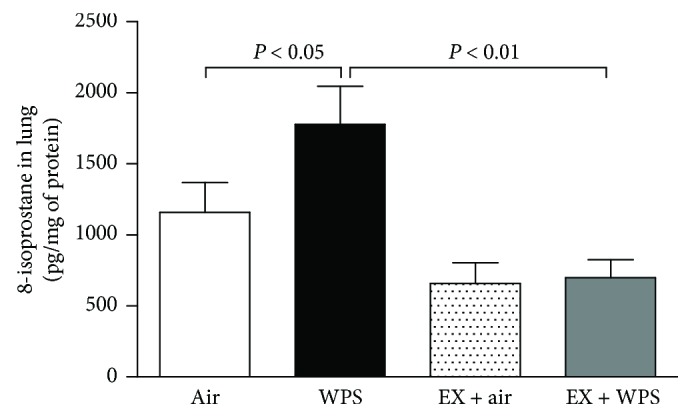
8-Isoprostane concentrations in lung homogenate, at the end of the 2-month-exposure period to water pipe smoke (WPS) or air with or without exercise (EX) training. Data are mean ± SEM (*n* = 5–6 in each group).

Figure 4 illustrates the concentrations of 8-isoprostane in lung homogenates after the subchronic exposure to WPS or air, with or without exercise training.

Compared with the air-exposed group, WPS exposure induced a significant increase in 8-isoprosatne concentration (*P* < 0.05). The latter effect was completely prevented in the exercise + WPS group compared with the WPS group (*P* < 0.01).

### 3.5. Lung DNA Damage

**Figure 5:**
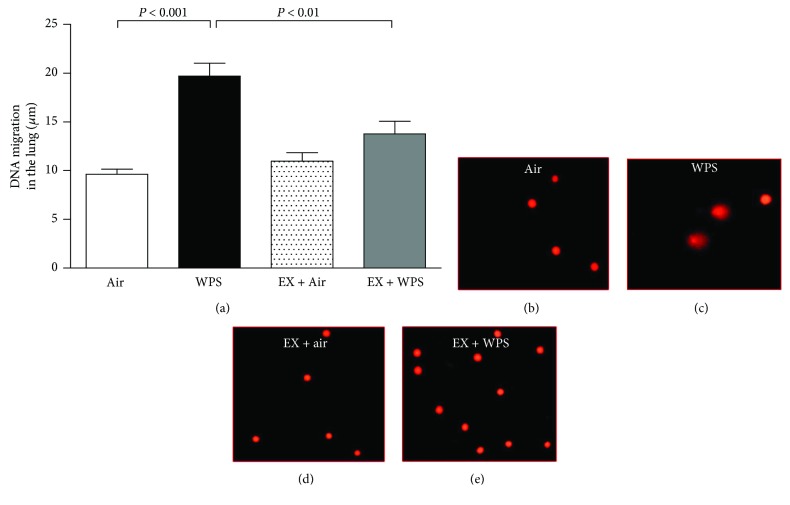
DNA migration (mm) in the lung tissues (a) evaluated by Comet assay, at the end of the 2-month-exposure period to water pipe smoke (WPS) or air with or without exercise (EX) training. Data are mean ± SEM (*n* = 5 in each group). Representative images illustrating the quantification of the DNA migration by the Comet assay under alkaline conditions, in control (b), WPS (c), EX + air (d), and EX + WPS (e).

Figure 5 illustrates the effect of subchronic WPS exposure on lung DNA damage and the influence of exercise training thereon. Compared with the air-exposed group, WPS exposure caused a significant increase in DNA migration (*P* < 0.001). The latter effect was significantly reversed in the exercise + WPS group compared with the WPS group (*P* < 0.01).

### 3.6. Western Blot Analysis for the Detection of NF-*κ*B and Nrf2

**Figure 6:**
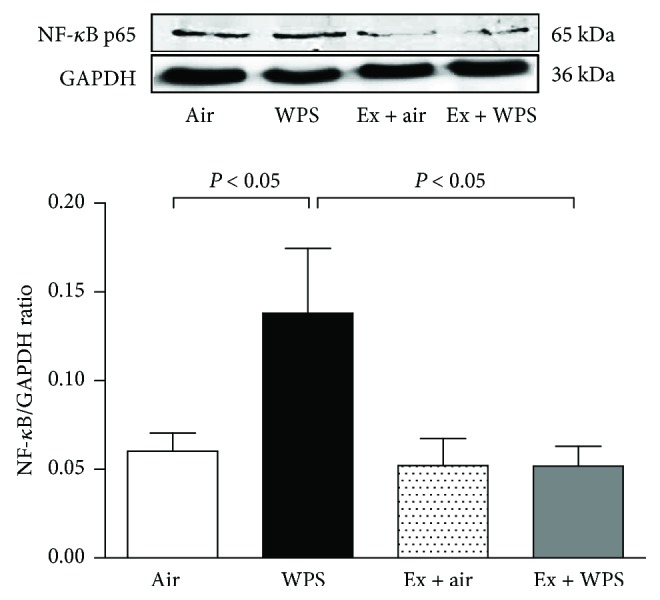
Western blot analysis and graphic representation of nuclear factor kappa-B (NF-*κ*B) protein levels in the lung tissues, at the end of the 2-month-exposure period to water pipe smoke (WPS) or air with or without exercise (EX) training. Data are mean ± SEM (*n* = 4–5 in each group).

The subchronic exposure to WPS caused an increase in the expression of NF-*κ*B (*P* < 0.05). Such effect was significantly prevented in the exercise + WPS group compared with the WPS group (*P* < 0.05) (Figure 6).

**Figure 7:**
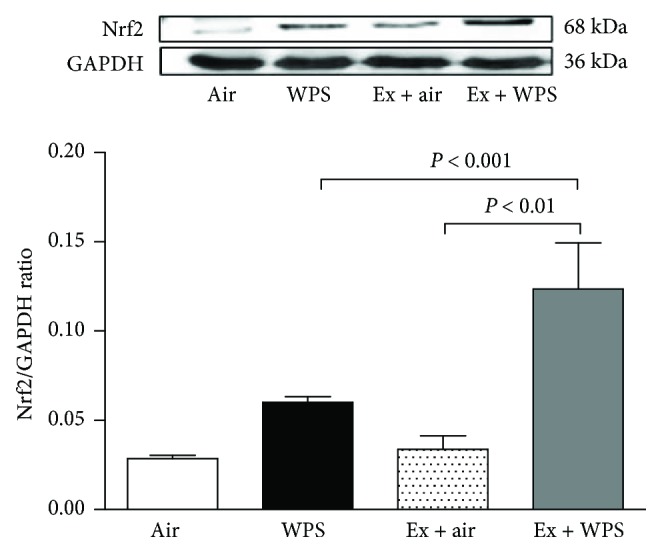
Western blot analysis and graphic representation of nuclear factor erythroid 2-related factor 2 (Nrf2) protein levels in the lung tissues, at the end of the 2-month-exposure period to water pipe smoke (WPS) or air with or without exercise (EX) training. Data are mean ± SEM (*n* = 4–5 in each group).

Figure 7 shows that, compared with the control group, the subchronic exposure to WPS induced a statistically insignificant increase of Nrf2 expression. The levels of expression of Nrf2 were significantly increased in the exercise + WPS versus WPS (*P* < 0.001) and the exercise + WPS versus exercise + air groups (*P* < 0.01).

## 4. Discussion

The current work provided experimental evidence that exercise training significantly mitigated subchronic pulmonary toxicity of WPS. We showed that regular exercise training alleviated WPS-induced airway resistance, inflammation, oxidative stress, and DNA damage via inhibiting NF-*κ*B and activating Nrf2 signalling pathways.

WPS is gaining extensive popularity all over the world in different populations [8]. A study has reported that in the USA, there are around 300 WPS bars situated in 2/3 of the states, regularly located close to colleges and universities [37]. Also, it has been reported that WPS could be a gateway to other forms of smoking including cigarette smoking, which could decline the progresses in tobacco reduction over the past decades [38]. Owing to the fact that the majority of water pipe smokers are also current or past smokers of cigarettes, clinical investigations on the adverse effects of WPS have described problems in investigating the sole effects of WPS [39]. Consequently, experimental research on this topic is important and necessary to uncover the possible mechanisms underlying the adverse effects of WPS and enable therapeutic or preventative strategies aiming at alleviating the pathophysiological effects of WPS.

Previous experimental studies have reported the positive impact of exercise training on cigarette smoke or particulate air pollution [40, 41]. However, as far as we are aware, no study has investigated the impact of exercise training on WPS-induced pulmonary impairment and the mechanisms underlying these effects.

The nose-only and whole body exposure systems are the two principal techniques currently used to study the impact of tobacco smoke and water pipe exposure in mice or rats [28, 42]. The shortcoming of using whole body exposure is that the animals may ingest nicotine or tar substances when cleaning their fur [28]. The nose-only exposure system avoids this problematic and most probably best resembles the human exposure circumstances [28, 43]. Moreover, we have recently reported that the levels of carboxyhemoglobin found in mice exposed to nose-only WPS were comparable with those reported in water-pipe smokers [23]. We have previously reported that acute (5 days) exposure to WPS induces lung inflammation but no change in airway resistance [44]. We have also reported that both lung inflammation and airway resistance were significantly increased following subacute (1 month) and chronic (6 months) exposures to WPS [14, 15]. In the present study, we showed that subchronic (2 months) exposure to WPS causes airway hyperresponsiveness to methacholine and that this effect was significantly prevented by regular exercise training. Moreover, we have also found that exercise training preserved the lung architecture and has significantly alleviated the interstitial and intra-alveolar infiltration of inflammatory cells and prevented the focal damage of alveolar septum induced by the subchronic exposure to WPS. It has been reported that aerobic physical training of moderate intensity attenuated the development of pulmonary emphysema and lung elastance induced by chronic (24 weeks) cigarette smoke exposure [40]. It has also been shown that regular aerobic exercise training exerts a protective effect against pulmonary inflammation induced by exposure to diesel exhaust particles (DEP) for 5 weeks [41].

Since several studies have reported that long-term regular aerobic exercise reduces oxidative stress and inflammation in animal models of chronic obstructive pulmonary disease [40] and asthma [45], we, presently, wanted to assess the effect of subchronic exposure to WPS on markers of inflammation and oxidative stress, and the possible protective effect of exercise training thereon. Our data show that subchronic exposure to WPS induced a significant increase of TNF*α* and IL-6. The latter proinflammatory cytokines were found to increase following 1 month of exposure to WPS. Both cytokines were shown to play an important role in the continuation of cigarette smoke-induced inflammation even following smoking cessation [46, 47]. Interestingly, here, we show that exercise training significantly reduced the increase of TNF*α* and IL-6 caused by WPS. Our findings are in agreement with a previous report which showed that exercise training prevented DEP-induced increase of TNF*α* and IL-6 in the lung [41]. Moreover, in this study, we have also measured the pulmonary concentrations of 8-isoprostane, a marker of lipid peroxidation. Our data show that exposure to WPS induced oxidative stress in lung tissue evidenced by a significant increase of 8-isoprostane in the lung tissue and that exercise training significantly reduced these effects. Our data is in line with a previous report which showed that exercise markedly prevented the increase in reactive oxygen species and 8-isoprostane expression in lung tissue caused by chronic exposure to cigarette smoke in mice [40].

It is well established that inflammation and oxidative stress result in cell membrane injury and DNA damage [48]. We have previously demonstrated that chronic exposure to WPS causes lung DNA damage [15]. Here, we show that subchronic exposure to WPS causes DNA damage and, remarkably, regular exercise training significantly averted this effect. This protective effect could be explained by the anti-inflammatory and antioxidant effects of exercise training. It has been previously reported that the use of gum acacia, a natural anti-inflammatory and antioxidant agent, averted the DNA damage in adenine-induced chronic kidney disease in rats [49].

NF-*κ*B, a nuclear transcription factor, plays an important role in several pathophysiological processes comprising inflammation, immune reaction, and apoptosis [50]. The activation of the NF-*κ*B signalling pathway induces the upregulations of proinflammatory cytokines including TNF*α*, IL-6, and IL-1*β* [50]. Studies in mouse models have reported that NF-*κ*B is an important mediator of many inflammatory disease states and strategies aiming at blocking this transcription factor may prevent inflammation-associated pulmonary diseases [50]. Also, it has been shown that inhibition of NF-*κ*B by geraniin, a natural compound with anti-inflammatory effect, [51] or penehyclidine hydrochloride, a selective anticholinergic agent, [52] alleviated lipopolysaccharide- (LPS-) induced acute lung injury in mice by blocking the production of proinflammatory cytokine. To investigate the mechanism by which exercise training exerts its beneficial effect against WPS-induced lung toxicity, we have assessed the effect of exercise training on NF-*κ*B activation. Our data show that WPS exposure induced a significant increase in the expression of NF-*κ*B and that exercise training significantly inhibited this effect. These results suggest that the protective effect exerted by exercise training is, at least partly, related to the inhibition of NF-*κ*B expression.

The transcription factor Nrf2 regulates the expression of antioxidant genes which regulate oxidative stress, xenobiotic metabolism and excretion, inflammation, and apoptosis, and several experimental studies showed the key role of Nrf2 activation to decrease oxidative stress and inflammation in animal models of pulmonary fibrosis, emphysema, acute lung injury, and asthma [53]. Our data show that subchronic exposure to WPS induced an insignificant increase in the expression of Nrf2 and the level of expression of this transcription factor was substantially and significantly increased in the exercise + WPS group. These data suggest that exercise training exerts its protective role against WPS-induced pulmonary toxicity by activating the Nrf2 signalling pathway. It has been recently reported in mice that Nrf2 expression is augmented in LPS-induced lung injury and the administration of geraniin caused an upregulation of Nrf2 expression and mitigated LPS-induced acute lung injury [51]. Additionally, another study reported that platycodin D, a natural compound with anti-inflammatory action, given i.p. to mice exerts a protective effect against cigarette smoke-induced pulmonary inflammation by averting inflammatory and oxidative response through activating the Nrf2 signalling pathway [54].

In conclusion, our data showed, for the first time, that aerobic exercise training significantly mitigated subchronic WPS-induced airway resistance, inflammation, oxidative stress, and DNA damage via inhibiting NF-*κ*B and activating Nrf2 signalling pathways. Further studies on the effect of different modalities of exercise on mice exposed to WPS for different durations are warranted. These preclinical findings may encourage further controlled studies in current water pipe smokers and in those who have stopped smoking to assess its clinical usefulness.
